# Identification of Allergic Epitopes of Soybean β-Conglycinin in Different Animal Species

**DOI:** 10.3389/fvets.2020.599546

**Published:** 2021-01-08

**Authors:** Yuan Zhao, Gaowa Naren, Jianan Qiang, Guixin Qin, Nan Bao, Mohammed Hamdy Farouk

**Affiliations:** ^1^Jilin Provincial Key Laboratory of Animal Nutrition and Feed Science, Key Lab of Animal Production, Product Quality and Security, College of Animal Science and Technology, Ministry of Education, Jilin Agricultural University, Changchun, China; ^2^Animal Production Department, Faculty of Agriculture, Al-Azhar University, Cairo, Egypt

**Keywords:** soybean, β-conglycinin, epitope, allergen, animal species

## Abstract

Soybean can cause allergy in both humans and animals. The herein study aims to identify the antigenic determinants (epitopes) of β-conglycinin that lead to allergy in different animal species (swine, bovine, and rats). The epitopes of β-conglycinin were identified through co-immunoprecipitation and mass spectrometry. The binding abilities of seven identified epitope peptides to allergic sera of three animal species were compared by ELISA and dot-blot techniques. Some epitope peptides could be recognized by the three animal allergic sera, while most epitopes showed some differences in binding abilities to the different animal sera. The strongest reaction using swine sera was detected with peptides α2, β2, and β3, but the biggest sensitive regions for bovine and rats were peptides α2, β1, and β4. Most epitopes of β-conglycinin exhibited different binding abilities to the three animal sera, in which the biggest sensitive regions were peptides α2, β2, and β3 for swine, but peptides α2, β1, and β4 were detected for bovine and rats.

## Introduction

Soybean is an important source of protein for both animals and humans worldwide. For example, the raw and processed forms of soybean represent ~63% of all protein sources. Such forms are commonly used as protein ingredients in human food and in different animal species feeds (1). Actually, the applicable amount of soybean meal in animal feeds (pets, poultry, swine, and bovine diets) exceeds 75% (2). However, soybean can cause allergy in both humans and animals, leading to some public concerns. At least 21 soybean allergens have been identified in humans (3), causing allergic symptoms including skin rash, gastrointestinal problems, rhinitis, asthma, hypotension, and anaphylaxis. Furthermore, soybean protein induces an allergic response in some animal species, such as swine (4–7), bovine (8, 9), rats (10), and dogs (11).

Allergic responses to soybeans may vary by species (2), breed (12), and age (13–15). For instance, newly weaned piglets suffering from soybean-induced allergy resulted in growth lag and malabsorption associated with intestinal histomorphology (16–19). Also, the Chinese indigenous pigs are less sensitive to soybean proteins than the Landrace pigs (12). Similar to piglets, preruminant calves exposed to soybean protein also exhibited allergic responses (8, 9). Also, the soybean-induced allergic rats exhibited incremental intestinal lymphocyte, deepened crypt, fallen villus, and lessened brush border enzyme activity that lead to malabsorption and even diarrhea (10). In general, soy allergy widely appears in young animals, but is rarely seen in adult animals (13–15). Therefore, understanding the potential difference among species to allergic responses in the juvenile stage to soybean protein is essential to minimize the allergic risk of soybean protein in different animal feeds.

The major soybean allergen β-conglycinin is made up of 67 kDa α-, 71 kDa α′-, and 50 kDa β-subunits (20). In order to establish the practical methods for allergen diagnosis, treatment, and even prevention and to provide soybean-breeding strategies for decreasing allergens, it is imperative to clarify soybean-allergic pathogenesis. Previous studies of β-conglycinin have mainly focused on IgG-binding epitopes of α-subunit in piglets (1), IgE-binding epitopes of α-subunit in humans (21, 22), and IgG-binding epitopes of β-subunit in multiple animals (e.g., pigs, dogs, rabbits, and fishes) (2). However, few studies have reported the epitopes of β-conglycinin in calves and rats which also are sensitive to soybean β-conglycinin. Moreover, the comparison of species-specific epitopes was only developed for β-subunit of β-conglycinin but not for all the three subunits of β-conglycinin.

Therefore, the objectives of this study are to identify the epitopes of β-conglycinin in different species (swine, bovine, and rats) and to compare the binding abilities of seven identified epitope peptides to allergic sera of such animal species. These results may further clarify the allergic difference of soy protein among animal species.

## Materials and Methods

### Animal Sera

To conform to national and international research standards, this study was conducted according to ethical principles governing the usage of animal samples in research studies of this nature. The permission to feed animals and to use samples was also acquired from the Ethical Clearance Committee of Jilin Agricultural University. The permission numbers for the use and care of animals are KT2016010 for swine, KT2016015 for bovine, and KT2016006 for rats. All of the experimental animals were managed according to the Guidelines for the Care and Use of Experimental Animals of Jilin Agricultural University.

Oral sensitization by means of diets was adopted in all animal species. Swine diets were formulated according to the National Research Council (23). The sensitized model of weaned Duroc × Landrace × Yorkshire piglets was founded as described by Sun et al. (4). During the sensitization stage, the piglets in the sensitized group were fed with an allergic diet using soybean, and the control diet was given on all the other days. The ingredients of the swine diets were described in our previous study (7). Briefly, the control diet contained 11.13% casein, 65.70% corn, 4.50% bran, 7.19% whey powder, 2.0% fish meal, 1.02% digested tankage, and 8.46% supplement (mineral, vitamins, amino acids, etc.), based on a dry matter basis. The allergic diet contained 30% peeled soybean meal, 5.00% soybean, 52.10% corn, 1.30% bran, and 11.6% supplement (mineral, vitamins, amino acids, etc.), based on a dry matter basis. The suckling preruminant Holstein calves were raised by an allergic diet consisting of a soybean meal which replaces 20% of the milk consumption during the sensitization stage by the method of Sun et al. (24). The weaned Sprague–Dawley (SD) rats were orally sensitized by soybean β-conglycinin according to the method of Guo et al. (25). During the sensitization stage, the rats in the sensitized group were orally administrated with 20 mg soybean β-conglycinin per day, based on a special diet without leguminous plants. The ingredients and nutrients of the rat diet without soybean ingredients were described in another previous study (26). Briefly, the rat diet contained 20% casein, 39.7% cornstarch, 13.2% dextrin, 7% corn oil, 5% cellulose, and 4.8% supplement (mineral, vitamins, amino acids, etc.), based on a dry matter basis. The animals in the control group were fed with a leguminous-free diet.

All the sensitized animals had higher β-conglycinin-specific IgG level. Furthermore, in the active cutaneous anaphylaxis test (piglets and calves) or the passive cutaneous anaphylaxis test (rats), the sensitized animals showed that the diameter of red flares or bluing at the injection sites was >5 mm in any direction. After successfully establishing the sensitized models of the three animal species, the bloods of allergic animals were collected. A 10-ml sample of blood was withdrawn from the precaval vein of each piglet or calves using a vacuum tube. A 3-ml sample of blood was drawn from the heart from each ether-anesthetized mice using the vacuum tube. The IgG in the allergic sera of different species was purified by protein A affinity chromatography (GE Healthcare). The β-conglycinin-specific IgG titer of different species in the allergy group was detected by the ELISA method. The IgG of animals in the control group was used as the negative control. Phosphate buffer solution (PBS) was used as the blank control. β-Conglycinin-specific IgG titers were determined by the method of Zhang et al. (27). β-Conglycinin-specific IgG was positive (+), when the optical density (OD_450_) value ratio of sample to negative control was >2:1; otherwise, it was negative (–). The OD_450_ value was recorded using the UV-2102PC ultraviolet–visible spectrophotometer (UNICO Instrument Co., Ltd, Shanghai, China).

### *In vitro* Enzymatic Hydrolysis of β-Conglycinin

β-Conglycinin was sufficiently hydrolyzed according to our previous method (28). Briefly, β-conglycinin was hydrolyzed by pepsin (enzyme/substrate, 1:1) for 30 min and then by trypsin (enzyme/substrate, 1:10) for 15 min. At the end of the final hydrolysis, the enzyme was inactivated by heating (80°C) for 5 min.

### Co-immunoprecipitation

The co-immunoprecipitation (co-IP) experiment was conducted to separate all the β-conglycinin enzymatic hydrolysates that could specifically bind to the IgG in the allergic serum from different animal species. Such experiment was carried out using the Pierce Crosslink Co-Immunoprecipitation Kit (Pierce Biotechnology, Rockford, USA). The conditions of all buffers provided in the kit were conducted strictly in accordance with the manufacturer's instructions. First, 30 μg IgG from the allergic serum was immobilized onto an AminoLink Plus Coupling Resin on a rotator overnight at 4°C. Second, β-conglycinin enzymatic hydrolysates were solubilized using IP lysis/wash buffer at a final concentration of 7–8 mg/ml. Third, soluble fractions (100–500 μl) from hydrolysates (input) were incubated at 37°C for 120 min with IgG-immobilized beads. Fourth, beads were centrifuged at 1,000 × g for 1 min and washed three times with PBS before elution. Then, protein samples (epitope peptides of enzymatic hydrolysates specially combined with IgG from the allergic serum) were eluted by the elution buffer.

To determine the effectiveness of elution, we analyzed the partial protein contents of the samples before and after elution by bicinchoninic acid kit for protein determination (BCA kit, Thermo, Waltham, MA, USA).

### Mass Spectrometry

The β-conglycinin enzymatic peptides specifically combining to IgG in the allergic serum were analyzed using an electrospray ionization tandem mass spectrometry LC-MS-MS (Q-E MS) by the Beijing Huada Protein Innovation Co., Ltd (Beijing, China). Mascot protein scores (based on combined MS and MS/MS spectra) >22 were considered statistically significant (*p* < 0.05). The individual MS/MS spectrum with a statistically significant (confidence interval > 95%) iron score was accepted.

The peptides were dissolved in 0.1% formic acid (solvent A) and directly loaded onto an Acclaim PepMap C18-reversed phase column (75 μm × 2 cm, 3 μm, 100 , Thermo Scientific) and separated with reversed phase C18 column (75 μm × 10 cm, 5 μm, 300 , Agela Technologies) mounted onto a Dionex ultimate 3,000 nano LC system. Peptides were eluted using the following gradients: 0–6 min 5–8% buffer B; 6–40 min 8–30% buffer B; 40–45 min 30–60% buffer B; 45–48 min 60–80% buffer B; 48–56 min 80% buffer B; 56–58 min 80–5% buffer B (decreasing to 5%); and 58–65 min 5% buffer B at a flow rate of 400 nl min^−1^ combined with a Q-Exactive mass spectrometer (Thermo Fisher Scientific, MA, USA). The eluates were directly entered for Q-Exactive MS (Thermo Fisher Scientific, Waltham, MA, USA), setting in a positive ion mode and data-dependent manner with full MS scan from 350 to 2,000 *m*/*z*, full scan resolution at 70,000, MS/MS scan resolution at 17,500, MS/MS scan with minimum signal threshold 1E+5, and isolation width at 2 Da. To evaluate the performance of this mass spectrometry on the labeled samples, using two MS/MS acquisition modes, higher collision energy dissociation (HCD) was employed. Also, to optimize the MS/MS acquisition efficiency of HCD, normalized collision energy (NCE) was systemically examined with the value of 28%, and then increased to 20%.

The peptides were characterized by manually interpreting the MS data with the help of the Mascot search engine version 2.3.01 (Matrix Science, Boston, MA, USA) and the integrated false discovery rate (FDR) analysis function. The previously identified amino acid sequences were matched to the known protein (soybean β-conglycinin) in the publicly available database (UniProt protein database). The previously identified amino acid sequences of soybean β-conglycinin (P13916, P11827, and P25974) were found in the UniProt protein database. All the MS experiments were tested and analyzed in triplicate.

The identified peptides were submitted to the Immune Epitope Database and Analysis Resource (http://www.iedb.org, the National Institute of Allergy and Infectious Diseases, Bethesda, MD, USA) to match with the epitopes of the allergen β-conglycinin previously described.

### ELISA and Dot-Blot

Based on the herein results and other previous studies (1, 2), we selected seven epitope peptides that could bind to IgG from the allergic serum of pigs. These identified epitope peptides with purity of over 90% were synthesized by the Beijing Huada Protein Innovation Co., Ltd. (Beijing, China).

The seven synthesized epitope peptides were, respectively, incubated in an ELISA plate at 37°C, respectively. The ELISA assay was performed in the same way as described in the study of Taliercio and Kim (29). Briefly, the seven synthesized epitope peptides were used as antigen, and then the optimal dilution ratio of allergic sera of different species was determined when the OD was about 1.0. The allergic sera of pigs (diluted 1:100), calves (diluted 1:1,000), and rats (diluted 1:10) were used as the primary antibody. To identify which kind of sera had specific antibodies against the peptides on the ELISA plates, we used horseradish peroxidase (HRP)-labeled species-specific antibodies against the IgG of swine (bs-0309R-HRP, Bios, Beijing, China), bovine (SE233, Solarbio Science & Technology, Beijing, China), and rats (CW0102S, ComWin Biotech, Beijing, China).

A dot-blot experiment was carried out to test the binding ability of the seven different epitope peptides to the allergic serum. Nitrocellulose membranes (0.45 μm pore size) were immersed in double-distilled water for 10 min. When the membranes were semidry, the seven dissolved epitope peptides with carbonate buffered saline (CBS, pH 9.6) were spotted in the painted circle by a Pap Pen (Solarbio, Beijing, China) on the membranes and then were left to dry at room temperature (5 μg for each dot). The spotted membranes were incubated for 2 h in PBST (0.1% Tween 20 in 0.01 M pH 7.4 phosphate-buffered saline) containing 2.5% non-fat dry milk (NFDM) to block the non-specific binding sites at 37°C. The membranes were then incubated with the allergic sera of piglets, calves, and rats (diluted 1:100, 1:1,000, and 1:10 with PBST/2.5% NFDM as the primary antibody, respectively). Following overnight incubation at room temperature, the membranes were then washed for 5 min with PBST three times and further incubated in PBST/2.5% NFDM for 1 h containing horseradish peroxidase (HRP)-labeled species-specific antibodies against the IgG of swine (Bios, Beijing, China), bovine (Solarbio Science & Technology, Beijing, China), and rats (ComWin Biotech, Beijing, China) at 37°C. Then, the membranes were washed three times with PBST (10 min each) followed by two washes with PBS (5 min each) and incubated with 3,3′-diaminobenzidine (DAB) horseradish peroxidase chromogenic substrate for 3 min in the darkness. Finally, the reaction was stopped by the removal of the strips from the chromogenic solution and washed in double-distilled water.

### Statistical Analysis

The optical density (OD) values in ELISA were analyzed using the general linear model of Statistical Package for Social Sciences version 11.5 (SPSS Inc., Chicago, IL, USA). The epitope peptide treatment was the independent factor in the statistical model. The results were expressed as mean ± standard deviation (SD). Duncan's multiple range tests were performed to test the differences among the means. Statements of statistical significance were based upon *p* ≤ 0.05.

## Results

### β-Conglycinin-Specific IgG Titer of Different Species

The OD values of β-conglycinin-specific IgG in the allergy group of piglet, calf, and rat are listed in Table 1. The OD values of β-conglycinin-specific IgG in the control groups (negative control) of pig, calf, and rat were very low (about 0.034~0.046). The levels of β-conglycinin-specific IgG in the allergy groups were higher compared with those of the control groups. The β-conglycinin-specific IgG titers of piglet, calf, and rat were 1:1,280, 1:10,240, and 1:160.

**Table 1:** β-Conglycinin-specific IgG titer of different animal species.

**Dilution ratio**	**Pig**		**Calf**		**Rat**	
	**Ave OD_**450**_**	**Positive/negative**	**Ave OD_**450**_**	**Positive/negative**	**Ave OD_**450**_**	**Positive/negative**
10	1.140 ± 0.101	+	1.249 ± 0.111	+	0.959 ± 0.091	+
20	0.993 ± 0.089	+	1.153 ± 0.095	+	0.806 ± 0.081	+
40	0.922 ± 0.081	+	0.866 ± 0.078	+	0.585 ± 0.053	+
80	0.762 ± 0.067	+	0.728 ± 0.069	+	0.378 ± 0.032	+
160	0.716 ± 0.056	+	0.477 ± 0.041	+	0.264 ± 0.021	+
320	0.473 ± 0.045	+	0.442 ± 0.039	+	0.059 ± 0.004	–
640	0.435 ± 0.039	+	0.398 ± 0.037	+	0.030 ± 0.002	–
1,280	0.192 ± 0.017	+	0.376 ± 0.029	+	0.019 ± 0.001	–
2,560	0.131 ± 0.009	–	0.330 ± 0.021	+	0.018 ± 0.001	–
5,120	0.040 ± 0.003	–	0.153 ± 0.011	+	0.003 ± 0.000	–
10,240	0.016 ± 0.002	–	0.134 ± 0.010	+	0.001 ± 0.000	–
20,480	0.011 ± 0.001	–	0.039 ± 0.004	–	0.001 ± 0.000	–
40,960	0.002 ± 0.000	–	0.015 ± 0.001	–	0.000 ± 0.000	–

### Identification of Antigenic Epitopes of β-Conglycinin Using Different Species Sera

All the identified epitope peptides in this study were submitted to bioinformatics analyses in the Immune Epitope Database and Analysis Resource (http://www.iedb.org). The co-IP and MS methods were performed to identify the allergic antigen epitopes of β-conglycinin using different species' sera. The identified epitopes of the α-, α′- and β-subunits of β-conglycinin are listed in Tables 2–4, respectively. The epitope peptides (189–197, 219–227, 287–305, 306–318, 309–318, and 422–429) of the α-subunit, the epitope peptides (225–231 and 240–247) of the α′-subunit, and the epitope peptides (63–70, 103–113, 126–144, 148–157, and 256–263) of the β-subunit were identified as commonly shared (allergic antigen) epitopes in all three animal species (swine, bovine, and rat). However, the epitope peptides (319–339, 340–349, 371–381, and 435–458) of the α-subunit, the epitope peptides (340–353, 374–383, 406–416, and 469–492) of the α′-subunit, and the epitope peptides (158–194, 195–215, and 269–292) of the β-subunit were only recognized by pig sera, whereas the epitope peptides (599–605) of the α′-subunit were only recognized by rat sera. Furthermore, the epitope peptides (398–409 and 410–418) of the α-subunit, the epitope peptides (340–353) of the α′-subunit, and the epitope peptides (28–37, 114–125, and 195–204) of the β-subunit could bind to both bovine and rat allergic sera.

**Table 2:** Amino acid sequence of the identified epitope peptides of the α-subunit of β-conglycinin using different animal sera.

**Amino acid sequence of the identified epitopes**	**Protein information in the UniProt database**			**Peptides recognized by different species sera**			**Blast in the epitope database**			
	**Accession no**.	***m*/*z***	**Score**	**Pig**	**Bovine**	**Rat**	**Matched degree**	**Epitope no**.	**Host**	**References**
NPFLFGSNR 189–197	P13916	526.270	37.240	+	+	+	Substring	32585 181406	Pig	Fu et al. (1) Sun et al. (22)
SPQLQNLR 219–227	P13916	478.271	38.940	+	+	+	Substring	38973	Pig	Fu et al. (1)
VPSGTTYYVVNPDNNENLR 287–305	P13916	1,076.520	83.750	+	+	+	Blast-90%	181524	Human	Sun et al. (22)
LITLAIPVNKPGR 306–318	P13916	696.442	40.060	+	+	+	Substring	181524	Human	Sun et al. (22)
FESFFLSSTEAQQSYLQGFSR 319–339	P13916	1,230.083	130.210	+	–	–	Blast-90%	181316	Human	Sun et al. (22)
NILEASYDTK 340–349	P13916	577.292	47.970	+	–	–	No match			
LQESVIVEISK 371–381	P13916	622.859	60.320	+	–	–	No match			
TISSEDKPFNLR 398–409	P13916	703.870	50.250	–	+	+	Blast-90%	181307 181455	Human	Sun et al. (22)
SRDPIYSNK 410–418	P13916	540.277	46.830	–	+	+	Substring	181455	Human	Sun et al. (22)
FFEITPEK 422–429	P13916	505.764	52.130	+	+	+	Substring	181292 181455	Human	Sun et al. (22)
DLDIFLSIVDMNEGALLLPHFNSK 435–458	P13916	906.473	59.050	+	–	–	No match			

**Table 3:** Amino acid sequence of the identified epitope peptides of α′ subunit of β-conglycinin using three different animal sera.

**Amino acid sequence of the identified epitopes**	**Protein information in the UniProt database**			**Peptides recognized by different animal sera**		
	**Accession no**.	***m*/*z***	**Score**	**Pig**	**Bovine**	**Rat**
NQYGHVR 225–231	P11827	437.219	29.780	+	+	+
SQQLQNLR 240–247	P11827	493.773	45.880	+	+	+
MITLAIPVNKPGR 340–353	P11827	475.948	58.600	+	+	+
NILEASYDTK 374–383	P11827	577.292	47.970	+	–	–
LQESVIVEISK 406–416	P11827	622.859	60.320	+	–	–
DLDVFLSVVDMNEGALFLPHFNSK 469–492	P11827	908.460	62.610	+	–	–
DIENLIK 599–605	P11827	422.744	28.990	–	–	+

**Table 4:** Amino acid sequence of the identified epitope peptides of the β-subunit of β-conglycinin using different animal sera.

**Amino acid sequence of the identified epitopes**	**Protein information in the UniProt database**			**Peptides recognized by different species sera**			**Blast in the epitope database**			
	**Accession no**.	***m*/*z***	**Score**	**Pig**	**Bovine**	**Rat**	**Matched degree**	**Epitope no**.	**Host**	**References**
EDENNPFYFR 28–37	P25974	665.789	58.050	–	+	+	Substring	227798	Fish	Taliercio et al. (2)
SPQLENLR 63–70	P25974	478.762	30.550	+	+	+	Substring	227764	Pig	Taliercio et al. (2)
AILTLVNNDDR 103–113	P25974	622.336	37.130	+	+	+	Substring	227802	Rabbit	Taliercio et al. (2)
IPAGTTYYLVNPHDHQNLK 126–144	P25974	727.709	61.180	+	+	+	Substring	227772	Rabbit	Taliercio et al. (2)
LAIPVNKPGR 148–157	P25974	532.834	58.560	+	+	+	Substring	227790	Rabbit	Taliercio et al. (2)
YDDFFLSSTQAQQSYLQGFSHNILETSFHSEFEEINR 158–194	P25974	1,104.519	25.040	+	–	–	Substring	227820	Pig	Taliercio et al. (2)
VLFGEEEEQRQQEGVIVELSK 195–215	P25974	816.089	35.600	+	–	–	Blast-90%	227835 227858	Rabbit Rabbit	Taliercio et al. (2)
VLFGEEEEQR 195–204	P25974	618.301	55.880	—	+	+	No match			
FFEITPEK 256–263	P25974	505.764	52.130	+	+	+	Substring	227769 227865	Pig Pig, rabbit	Taliercio et al. (2)
DLDIFLSSVDINEGALLLPHFNSK 269–292	P25974	886.477	84.930	+	–	–	Substring	227754 227803	Pig, dog, fish Pig, dog, fish	Taliercio et al. (2)

Some of the amino acid sequences of the identified epitope peptides in this study, consistent with the known epitopes of β-conglycinin, are shown in Tables 2–4. In the α-subunit of β-conglycinin, the epitope peptides (189–197, 224–232, 306–318, 410–418, and 422–429) were substring to the known epitopes, and the epitope peptides (287–305, 319–339, and 398–409) were blast-90% to the known epitopes. The blast of the α′-subunit of β-conglycinin could not be conducted considering the information in the epitope database. Regarding the β-subunit of β-conglycinin, most of the identified epitope peptides except “VLFGEEEEQR” were entirely or partly consistent with the known epitopes.

### Comparison of the Sensitivity of the Seven Identified Allergic Antigen Epitopes of β-Conglycinin Binding to Different Animal Sera

**Figure 1:**
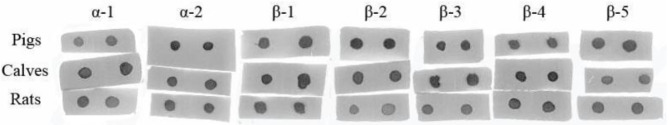
Comparison of the binding abilities of epitopes to the different animal species' allergic sera by dot-blot.

To compare the sensitive region among different species, seven antigenic epitopes of β-conglycinin were selected and synthesized to test the species-specific IgG-binding ability of peptides by ELISA and dot-blot as listed in Table 5. The results of ELISA were quite close to the results of the dot-blot. Using the two approaches, the strongest reaction to piglet sera was detected with peptides α2, β2, and β3, whereas peptides α1 and β1 showed relatively weak reactions (Table 6, Figure 1). However, the sensitive region for calves and rats was different from that for piglets. For example, the intensity of peptides α2, β1, and β4 was the strongest, and the weakest sensitivity was for peptides β2, β3, and β5.

**Table 5:** Synthetic antigen epitope peptides of β-conglycinin.

**Peptide name**	**Amino acid sequence of peptides**	**Antigen**
α1	NPFLFGSNR 189–197	α-Subunit of β-conglycinin
α2	SPQLQNLR 219–227	
β1	SPQLENLR 63–70	β-Subunit of β-conglycinin
β2	LLQRF 55–59	
β3	QGFSHNILETSFHSEF 173–189	
β4	FFEITPEK 256–263	
β5	DLDIFLSSVDINEGALLLPHFNSK 269–292	

**Table 6:** Comparison of the binding abilities of allergic epitopes among different animal sera by ELISA.

**Species**	**β-Conglycinin synthetic epitope peptides**							***p*-value**
	**α1**	**α2**	**β1**	**β2**	**β3**	**β4**	**β5**	
Pig	0.216 ± 0.004^a^	0.463 ± 0.004^c^	0.274 ± 0.008^a^	0.522 ± 0.005^c^	0.477 ± 0.011^c^	0.303 ± 0.005^b^	0.362 ± 0.004^b^	<0.001
Calf	0.267 ± 0.005^b^	0.271 ± 0.021^bc^	0.362 ± 0.004^c^	0.220 ± 0.013^a^	0.249 ± 0.007^ab^	0.306 ± 0.049^c^	0.208 ± 0.004^a^	<0.001
Rat	0.258 ± 0.003^b^	0.298 ± 0.003^c^	0.289 ± 0.005b^c^	0.192 ± 0.007^a^	0.217 ± 0.003^a^	0.316 ± 0.003^c^	0.223 ± 0.004^ab^	<0.001

*Means in the same row followed by different superscripts are significantly different (p < 0.05)*.

## Discussion

The allergy-causing epitopes of the α-, α′-, and β-subunits of β-conglycinin were identified in different animal species (swine, bovine, and rats) by allergic sera IgG in this study, because the standard IgE in animals and anti-swine/bovine IgE antibodies are not available (30). Actually, sera IgG binding has been successfully employed by many researchers in allergenicity study using different animal models (29, 31–33). In this study, the levels of β-conglycinin-specific IgG in piglet, calf, and rat of the allergy group rose up after ingestion of a soybean meal, which indicated that soybean has successfully induced allergy models in the three animal species.

Soybean allergens are sensitized *via* the major route of food/feed ingestion. Most proteins are digested into amino acids, small peptides, or both forms to facilitate absorption by gastrointestinal mucosa (34). Nevertheless, some allergic proteins can partially resist proteolysis along the gastrointestinal tract, such as immnunoreactive fragments that induce sensitization after their absorption (35, 36). Thus, some epitope peptides may exist in the undegraded fragments. Therefore, this study used allergic sera to disclose the allergy-causing epitope peptides through co-IP and MS, similar to the method adopted by Verissimo da Costa et al. (37).

Experimental results indicated that the allergic sera of three animal species could recognize the epitope peptides in each subunit of β-conglycinin. These data confirm that the α-, α′-, and β-subunits of β-conglycinin are antigenic across different animal species (38). This can be explained by the fact that the genes encoding the α-, α′-, and β-subunits of β-conglycinin exhibited extensive sequence homology (39, 40). As such, some antigenic epitopes with the same amino acid sequences in different subunits were also identified in the current study. Certainly, the structural differences among closely related protein subunits have significant effects on the sensitive regions of antigens. So, not all peptides with the same amino acid sequences were recognized by animal sera.

The common antigenic regions of β-conglycinin in all three animal species were determined in this study, which were also identified by others. The identified epitope peptides “NPFLFGSNR (189–197)” and “SPQLQNLR (219–227)” of the α-subunit and “SPQLENLR (63–70), YDDFFLSSTQAQQSYLQGFSHNILETSFHSEFEEI (158–194), FFEITPEK (256–263), and DLDIFLSSVDINEGALLLPHFNSK (269–292)” of the β-subunit recognized by pig allergic sera in the current study are consistent with the findings reported previously (1, 2). Among them, the FFEITPEK (256–263) and DLDIFLSSVDINEGALLLPHFNSK (269–292) were also detected in dog, rabbit, and fish sera (2), which stood out as important antigenic regions. Some epitope peptides are substring to the known epitope in the database, although the antibody types used differ from our study. The peptides “LITLAIPVNKPGR (306–318), SRDPIYSNK (410–418), and FFEITPEK (422–429)” of the α-subunit were also recognized by anti-human IgE antibody (22). The peptides “EDENNPFYFR (28–37)” of the β-subunit were also identified by anti-fish IgM (2) or anti-rabbit IgG antibodies (2). Notably, some epitope peptides exhibited binding ability to different animal species' allergic sera, and especially, they also could bind to human allergic sera. Those common epitope peptides act as important antigens. The identification of allergic epitopes can provide valuable information for exploring immunotherapy for soybean-caused allergy in humans and animals and for soybean breeding, targeting on reducing its allergic responses in the animal production sector.

The most identified epitope peptides in this study showed some differences in their ability of binding to the allergic sera of three different animal species. A similar study of the binding ability of allergy-causing epitope in different species was also conducted, and the difference among species was also verified (2). Seven synthetic epitope peptides were used to compare their immunoreactivities with three animal species' sera. Such epitope peptides exhibited different immunoreactivities among epitope peptides and animal species. Some peptides are easily recognized by specific animals, which is in agreement with a previous study (2). Peptides α2, β2, and β3 appeared to be more sensitive to pigs, but peptides α2, β1, and β4 exhibited strong ability to calf and rat sera. The disparate binding ability of antigenic epitopes could contribute to further clarify the allergic difference of soy proteins to various animal species.

In conclusion, the epitope peptides hydrolyzed from the three subunits of β-conglycinin probably causing allergy in animals were identified by using swine, bovine, and rat allergic sera. These common antigenic regions may be inactive in soybean breeding. Most of the identified epitope peptides in this study showed some difference in the ability of binding to the allergic sera of the three different animal species. The synthetic peptides α2, β2, and β3 exhibited strong binding ability to pig sera, but peptides α2, β1, and β4 showed easier reaction to calf or rat sera. Variants of the common antigenic regions will be useful in the optimization of soy proteins for different animal species.

## Data Availability Statement

The datasets presented in this study can be found in online repositories. The names of the repository/repositories and accession number(s) can be found in the article/Supplementary Material.

## Ethics Statement

The animal study was reviewed and approved by Ethical Clearance Committee of Jilin Agricultural University, Jilin Agricultural University, Changchun 130118, P.R. China.

## Author Contributions

YZ: study design, financial support, and manuscript writing. GN and JQ: performing the experiment and data collection. NB and GQ: statistical analysis of the data. MF: manuscript modification. All authors have read and agreed to the published version of the manuscript.

## Conflict of Interest

The authors declare that the research was conducted in the absence of any commercial or financial relationships that could be construed as a potential conflict of interest.

## References

[1] Fu CJ Jez JM Kerley MS Allee GL Krishnan HB. Identification, characterization, epitope mapping, and three-dimensional modeling of the alpha-subunit of beta-conglycinin of soybean, a potential allergen for young pigs. J Agric Food Chem. (2007) 55:4014–20. 10.1021/jf070211o 17439152

[2] Taliercio E Loveless TM Turano MJ Kim SW. Identification of epitopes of the β subunit of soybean β-conglycinin that are antigenic in pigs, dogs, rabbits and fish. J Agric Food Chem. (2014) 94:2289–94. 10.1002/jsfa.6556 24415270

[3] Babiker EE Hiroyuki A Matsudom N Iwata H Ogawaet T Effect of polysaccharide conjugation or transglutaminase treatment on the allergenicity and functional properties of soy protein. J Agric Food Chem. (1998) 46:866–71. 10.1021/jf9705072

[4] Sun P Li D Li Z Dong B Wang F. Effects of glycinin on IgE-mediated increase of mast cell numbers and histamine release in the small intestine. J Nutr Biochem. (2008) 19:627–33. 10.1016/j.jnutbio.2007.08.007 18280135

[5] Chen F Hao Y Piao XS Ma X Wu GY Qiao SY. Soybean-derived beta-conglycinin affects proteome expression in pig intestinal cells *in vivo* and *in vitro*. J Anim Sci. (2011) 89:743–53. 10.2527/jas.2010-3146 21057091

[6] Yi D Hou Y Mei H Wang L Hu CA Wu GY. β-conglycinin enhances autophagy in porcine enterocytes. Amino Acids. (2017) 49:203–7. 10.1007/s00726-016-2352-7 27761755

[7] Chang M Zhao Y Qin G Zhang XD. Fructo-oligosaccharide alleviates soybean-induced anaphylaxis in piglets by modulating gut microbes. Front Microbiol. (2018) 9:2769. 10.3389/fmicb.2018.02769 30524396 PMC6256172

[8] Lallés JP Dréau D Salmon H Toullec R. Identification of soya bean allergens and immunemechanisms of dietary sensitivities in preruminant calves. Res Vet Sci. (1996) 60:111–6. 10.1016/S0034-5288(96)90003-X 8685530

[9] Lallès JP Tukur HM Salgado P Mills EC Morgan MR Quillien L. Immunochemical studies on gastric and intestinal digestion of soybean glycinin and β-conglycinin *in vivo*. J Agric Food Chem. (1999) 47:2797–806. 10.1021/jf980882+ 10552568

[10] Guo PF Piao XS Cao YH Ou DY Li DF. Recombinant soybean protein β-conglycinin α'-subunit expression and induced hypersensitivity reaction in rats. Int Arch Allergy Immunol. (2008) 145:102–10. 10.1159/000108135 17823539

[11] Olivry T Bizikova P. A systematic review of the evidence of reduced allergenicity and clinical benefit of food hydrolysates in dogs with cutaneous adverse food reactions. Vet Dermatol. (2010) 21:32–41. 10.1111/j.1365-3164.2009.00761.x 19552700

[12] Qin G Xu L Jiang H van der Poel A Bosch M Verstegen M The effects of Chinese and Argentine soybeans on nutrient digestibility and organ morphology in Landrace and Chinese Min pigs. Asian Austral J Anim Sci. (2002) 15:555–64. 10.5713/ajas.2002.555

[13] Aoyama T Kohno M Saito T Fukui K Takamatsu K Yamamoto T. Reduction by Phytate-reduced Soybean β-conglycinin of plasma triglyceride level of young and adult rats. Biosci Biotechnol Biochem. (2001) 65:1071–5. 10.1271/bbb.65.1071 11440119

[14] Wang T Qin G Zhao Y Sun Z Comparative study on the stability of soybean (Glycine max) β-conglycinin *in vivo*. Food Agric Immunol. (2009) 20:295–304. 10.1080/09540100903203004

[15] Wang T Qin G Sun Z Zhao Y Comparative study on the residual rate of immunoreactive soybean glycinin (11S) in the digestive tract of pigs of different ages. Food Agric Immunol. (2010) 21:201–8. 10.1080/09540100903563597

[16] Li DF Nelssen JL Reddy PG Blecha F Klemm R Goodband RD. Interrelationship between hypersensitivity to soybean proteins and growth performance in early-weaned pigs. J Anim Sci. (1991) 69:4062–9. 10.2527/1991.69104062x 1778820

[17] Friesen KG Goodband RD Nelssen JL Blecha F Reddy DN Reddy PG. The effect of pre and post weaning exposure to soybean meal on growth performance and on the immune response in the early-weaned pig. J Anim Sci. (1993) 71:2089–98. 10.2527/1993.7182089x 8376233

[18] Kim SW van Heugten E Ji F Lee CH Mateo RD. Fermented soybean meal as a vegetable protein source for nursery pigs: I. Effects on growth performance of nursery pigs. J Anim Sci. (2010) 88:214–24. 10.2527/jas.2009-1993 19783703

[19] Huang Q Xu H Yu Z Gao P Liu S. Inbred Chinese Wuzhishan (WZS) minipig model for soybean glycinin and β-conglycinin allergy. J Agric Food Chem. (2010) 58:5194–8. 10.1021/jf904536v 20369852

[20] Holzhauser T Wackermann O Ballmer-Weber BK Bindslev-Jensen C Scibilia J Perono-Garoffo L. Soybean (Glycine max) allergy in Europe: Gly m 5 (beta-conglycinin) and Gly m 6 (glycinin) are potential diagnostic markers for severe allergic reactions to soy. J Allergy Clin Immunol. (2009) 123:452–8. 10.1016/j.jaci.2008.09.034 18996574

[21] Ogawa T Bando N Tsuji H Nishikawa K Kitamura K. Alpha-subunit of β-conglycinin, an allergenic protein recognized by IgE antibodies of soybean-sensitive patients with atopic-dermatitis. Biosci Biotechnol Biochem. (1995) 59:831–3. 10.1271/bbb.59.831 7787297

[22] Sun XL Shan XH Yan ZH Zhang YZ Guan L. Prediction and characterization of the linear IgE epitopes for the major soybean allergen β-conglycinin using immunoinformatics tools. Food Chem Toxicol. (2013) 56:254–60. 10.1016/j.fct.2013.02.014 23454299

[23] NRC Nutrient Requirements of Swine, Eleventh revised ed. Washington, DC: National Academy Press (2012).

[24] Sun ZW Qin GX Zhang QH Effect of steam-heated procedure on immunogenicity of major soybean antigenic proteins in whole fat soybean. Acta Nutrimenta Sinica. (2006) 28:522–5. 10.3321/j.issn:0512-7955.2006.06.020

[25] Guo PF Piao XS Ou DY Li DF Hao Y. Characterization of the antigenic specificity of soybean protein β-conglycinin and its effects on growth and immune function in rats. Arch Anim Nutr. (2007) 61:189–200. 10.1080/17450390701318358 17578261

[26] Zhang QY Zhao Y Qin GX Qiang JN Bao N Comparison of β-conglycinin and its major antigen epitope combined with small intestinal epithelial mucosal proteins in rats. Chin J Anim Nutr. (2019) 31:3235–41. 10.3969/j.issn.1006-267x.2019.07.035

[27] Zhang W Cheng BB Chen S Wang MS Jia RY Zhu DK Development and optimization of a double antibody sandwich ELISA for the detection of goose T cell surface CD8α molecule. J Integr Agric. (2016) 15:2363–8. 10.1016/S2095-3119(16)61345-X

[28] Zhao Y Qin GX Sun ZW Zhang B Wang T Stability and immunoreactivity of glycinin and β-conglycinin to hydrolysis in vitro. Food Agric Immunol. (2010) 21:253–63. 10.1080/09540101003758954

[29] Taliercio E Kim SW. Epitopes from two soybean glycinin subunits are antigenic in pigs. J Sci Food Agric. (2013) 93:2927–32. 10.1002/jsfa.6113 23426933

[30] Tryphonas H George A Elizabeth V Genevieve B. Animal models to detect allergenicity to foods and genetically modified products: workshop summary. Environ Health Perspect. (2003) 111:221–2. 10.1289/ehp.5701 12573909 PMC1241354

[31] Salgado P Freire J Ferreira R Seabra M Teixeira A Toullec R Legume proteins of the vicilin family are more immunogenic than those of the legumin family in weaned piglets. Food Agric Immunol. (2002) 14:51–63. 10.1080/09540100220137664

[32] Helm RM Ermel RW Frick OL. Nonmurine animal models of food allergy. Environ Health Perspect. (2003) 111:239–44. 10.1289/ehp.5705 12573913 PMC1241358

[33] Awazuhara H Kawai H Maruchi N. Major allergens in soybean and clinical significance of IgG4 antibodies investigated by IgE- and IgG4-immunoblotting with plasma from soybean sensitive patients. Clin Exp Allergy. (1997) 27:325–32. 10.1111/j.1365-2222.1997.tb00711.x 9088659

[34] Erickson RH Kim YS. Digestion and absorption of dietary protein. Annu Rev Med. (1990) 41:133–9. 10.1146/annurev.me.41.020190.001025 2184718

[35] Moreno FJ. Gastrointestinal digestion of food allergens: effect on their allergenicity. Biomed Pharmacother. (2007) 61:50–60. 10.1016/j.biopha.2006.10.005 17188456

[36] Asero R Antonicelli L. Does sensitization to foods in adults occur always in the gut? Int Arch Allergy Immunol. (2010) 154:6–14. 10.1159/000319203 20664272

[37] Verissimo da Costa GC Lery LM da Silva ML Moura H Peralta RHS von Krüger WM. The identification and characterization of epitopes in the 30-34 kDa *Trypanosoma cruzi* proteins recognized by antibodies in the serum samples of chagasic patients. J Proteomics. (2013) 80:34–42. 10.1016/j.jprot.2012.11.001 23159400

[38] Krishnan HB Kim WS Jang S Kerley MS. All three subunits of soybean β-conglycinin are potential food allergens. J Agric Food Chem. (2009) 57:938–43. 10.1021/jf802451g 19138084

[39] Maruyama N Katsube T Wada Y Oh MH Barba De La Rosa AP Okuda E. The roles of the N-linked glycans and extension regions of soybean β-conglycinin in folding, assembly and structural features. Eur J Biochem. (1998) 258:854–62. 10.1046/j.1432-1327.1998.2580854.x 9874256

[40] Harada JJ Barker SJ Goldberg RB. Soybean β-conglycinin genes are clustered in several DNA regions and are regulated by transcriptional and posttranscriptional processes. Plant Cell. (1989) 1:415–25. 10.1105/tpc.1.4.415 2562562 PMC159773

