# Supplementary material for: Identification of Allergic Epitopes of Soybean β-Conglycinin in Different Animal Species
Source: Front Vet Sci. 2021 Jan 8;7:599546. doi: 10.3389/fvets.2020.599546 (PMC7820328; doi:10.3389/fvets.2020.599546)
Supplement: Supplementary file 1 [file Table_1.DOCX]

**Table S1**. Ingredient composition and nutrient levels of the swine diets

| Ingredient composition（%） | Dietary treatment | |
| --- | --- | --- |
|  | Control diet | Allergy diet |
| Peeled Soybean Meal |  | 30.00 |
| Soybean |  | 5.00 |
| Casein | 11.13 |  |
| Zeolite | 0.80 | 0.80 |
| Corn | 65.70 | 52.10 |
| Bran | 4.50 | 1.30 |
| Limestine | 1.42 | 1.89 |
| Whey powder | 7.19 | 0.00 |
| Fish meal | 2.00 | 0.00 |
| Digested tankage | 1.02 | 0.00 |
| Salt | 0.65 | 1.00 |
| Sucrose | 2.55 | 1.20 |
| Vitamin-mineral premix* | 0.85 | 1.00 |
| Oil | 1.10 | 2.95 |
| Phosphate | 0.58 | 2.00 |
| Lysine | 0.29 | 0.45 |
| Threonine | 0.12 | 0.19 |
| Methionine | 0.10 | 0.12 |
| Total | 100 | 100 |
|  |  |  |
| Chemical analysis |  |  |
| Net energy(kJ/kg) | 10.24 | 10.24 |
| Crude protein (%) | 17.5 | 17.5 |
| Lysine (%) | 1.44 | 1.35 |
| Methionine (%) | 0.41 | 0.44 |
| Threonine (%) | 0.83 | 0.79 |
| Arginine (%) | 1.25 | 0.71 |
| Leucine (%) | 1.47 | 1.66 |
| Iso-Leucine (%) | 0.73 | 0.73 |
| Calcium (%) | 1.00 | 0.86 |
| Phosphorus (%) | 0.40 | 0.40 |
| Natrium (%) | 0.41 | 0.36 |
| Chlorine (%) | 0.62 | 0.53 |

*Premix provided per kilogram of complete diet: vitamin A, 45,000,000 IU; vitamin D3, 9,000,000 IU; vitamin E, 80,000 IU; vitamin K3, 5000 mg; vitamin B1, 8000 mg; vitamin B2, 20,000 mg; vitamin B6, 9000 mg; vitamin B12, 100 mg; nicotinamide, 100,000 mg; D-calpanate, 50000mg; folic acid, 4000 mg; D-biotin 500mg; Cu, 320 mg; Fe, 175mg; Zn, 125mg; Mn, 55mg; Se, 7.5mg and I, 20 mg.

Table S2. Composition and nutrient levels of the rat diet

| Items | Content（%） |
| --- | --- |
| Ingredient composition |  |
| Casein | 20.0000 |
| Corn starch | 39.7486 |
| Dextrin | 13.2000 |
| Sucrose | 10.0000 |
| Corn oil | 7.0000 |
| Cellulose | 5.0000 |
| Mineral premix 1 | 3.5000 |
| Vitamin premix 2 | 1.0000 |
| L-cystine | 0.3000 |
| Choline chloride | 0.2500 |
| TBHQ | 0.0014 |
| Total | 100.0000 |
| Nutrient levels 3 |  |
| GE (MJ/kg) | 15.76 |
| Trp (g /kg) | 2.10 |
| Thr (g /kg) | 6.70 |
| Met (g /kg) | 4.60 |
| Lys (g /kg) | 13.00 |
| AP (g /kg) | 0.30 |
| Ca (g /kg) | 0.50 |

1 Vitamin premix provided the following per kilogram of the diet: VA 0．004 IU， VD 0．001 IU， VE 0．000 075 IU， VK 0．9 mg， pantothenic acid 15 mg， riboflavin 6 mg， folic acid 2 mg， thiamine 5 mg， VB6 6 mg， biotin 0．2 mg， VB12 0．025 mg.

2 Mineral premix provided the following per kilogram of the diet: Zn ( ZnSO4·7H2O) 38 mg， Fe ( FeSO4 ·7H2O) 45 mg， Mn ( MnSO4·H2O) 10 mg， Cu ( CuSO4 ·5H2O) 6 mg， I ( KI) 0．2 mg.

3 GE was a calculated value, while the others were measured values
