# Supplementary material for: Identification of Allergic Epitopes of Soybean β-Conglycinin in Different Animal Species
Source: Front Vet Sci. 2021 Jan 8;7:599546. doi: 10.3389/fvets.2020.599546 (PMC7820328; doi:10.3389/fvets.2020.599546)
Supplement: Supplementary file 2 [file Table_2.DOCX]

| prot_hit_num | prot_acc | prot_score | prot_mass | prot_matches | prot_matches_sig | prot_sequences | prot_sequences_sig | pep_rank | pep_isunique | pep_exp_mz | pep_score | pep_expect | pep_seq |
| --- | --- | --- | --- | --- | --- | --- | --- | --- | --- | --- | --- | --- | --- |
|  |  |  |  |  |  |  |  |  |  |  |  |  |  |
| 1 | P13916 | 3906 | 70535 | 182 | 150 | 20 | 20 | 1 | 1 | 526.27 | 37.24 | 0.00019 | NPFLFGSNR |
| 1 | P13916 | 3906 | 70535 | 182 | 150 | 20 | 20 | 1 | 1 | 577.2919 | 47.97 | 1.60E-05 | NILEASYDTK |
| 1 | P13916 | 3906 | 70535 | 182 | 150 | 20 | 20 | 1 | 1 | 622.8593 | 60.32 | 9.30E-07 | LQESVIVEISK |
| 1 | P13916 | 3906 | 70535 | 182 | 150 | 20 | 20 | 1 | 0 | 505.7641 | 52.13 | 6.10E-06 | FFEITPEK |
| 1 | P13916 | 3906 | 70535 | 182 | 150 | 20 | 20 | 1 | 1 | 696.4419 | 40.06 | 9.90E-05 | LITLAIPVNKPGR |
| 1 | P13916 | 3906 | 70535 | 182 | 150 | 20 | 20 | 1 | 1 | 703.8701 | 50.25 | 9.40E-06 | TISSEDKPFNLR |
| 1 | P13916 | 3906 | 70535 | 182 | 150 | 20 | 20 | 1 | 1 | 1076.5199 | 83.75 | 4.20E-09 | VPSGTTYYVVNPDNNENLR |
| 1 | P13916 | 3906 | 70535 | 182 | 150 | 20 | 20 | 1 | 1 | 1230.0826 | 130.21 | 9.50E-14 | FESFFLSSTEAQQSYLQGFSR |
| 1 | P13916 | 3906 | 70535 | 182 | 150 | 20 | 20 | 1 | 1 | 478.2714 | 38.94 | 0.00013 | SPQLQNLR |
| 1 | P13916 | 3906 | 70535 | 182 | 150 | 20 | 20 | 1 | 1 | 906.4731 | 59.05 | 1.20E-06 | DLDIFLSIVDMNEGALLLPHFNSK |
| 1 | P13916 | 2450 | 70535 | 153 | 114 | 17 | 16 | 1 | 1 | 540.2772 | 46.83 | 2.10E-05 | SRDPIYSNK |
|  |  |  |  |  |  |  |  |  |  |  |  |  |  |
|  |  |  |  |  |  |  |  |  |  |  |  |  |  |
| 2 | P11827 | 1891 | 74565 | 97 | 75 | 14 | 14 | 1 | 1 | 493.7728 | 45.88 | 2.60E-05 | SQQLQNLR |
| 2 | P11827 | 1891 | 74565 | 97 | 75 | 14 | 14 | 1 | 1 | 475.9483 | 58.6 | 1.40E-06 | MITLAIPVNKPGR |
| 2 | P11827 | 1891 | 74565 | 97 | 75 | 14 | 14 | 1 | 0 | 577.2919 | 47.97 | 1.60E-05 | NILEASYDTK |
| 2 | P11827 | 1891 | 74565 | 97 | 75 | 14 | 14 | 1 | 0 | 622.8593 | 60.32 | 9.30E-07 | LQESVIVEISK |
| 2 | P11827 | 1891 | 74565 | 97 | 75 | 14 | 14 | 1 | 1 | 908.4601 | 62.61 | 5.50E-07 | DLDVFLSVVDMNEGALFLPHFNSK |
| 2 | P11827 | 63 | 74565 | 2 | 2 | 2 | 2 | 1 | 1 | 437.2192 | 29.78 | 0.0011 | NQYGHVR |
| 2 | P11827 | 1891 | 74565 | 97 | 75 | 14 | 14 | 1 | 1 | 422.7441 | 28.99 | 0.0013 | DIENLIK |
|  |  |  |  |  |  |  |  |  |  |  |  |  |  |
|  |  |  |  |  |  |  |  |  |  |  |  |  |  |
| 3 | P25974 | 328 | 50578 | 18 | 11 | 6 | 5 | 1 | 1 | 478.7619 | 30.55 | 0.00088 | SPQLENLR |
| 3 | P25974 | 328 | 50578 | 18 | 11 | 6 | 5 | 1 | 1 | 622.3363 | 37.13 | 0.00019 | AILTLVNNDDR |
| 3 | P25974 | 328 | 50578 | 18 | 11 | 6 | 5 | 1 | 1 | 1104.5188 | 25.04 | 0.0031 | YDDFFLSSTQAQQSYLQGFSHNILETSFHSEFEEINR |
| 3 | P25974 | 4218 | 50578 | 196 | 162 | 26 | 23 | 1 | 1 | 532.8336 | 58.56 | 1.40E-06 | LAIPVNKPGR |
| 3 | P25974 | 4218 | 50578 | 196 | 162 | 26 | 23 | 1 | 1 | 886.4765 | 84.93 | 3.20E-09 | DLDIFLSSVDINEGALLLPHFNSK |
| 3 | P25974 | 4218 | 50578 | 196 | 162 | 26 | 23 | 1 | 1 | 505.7641 | 52.13 | 6.10E-06 | FFEITPEK |
| 3 | P25974 | 328 | 50578 | 18 | 11 | 6 | 5 | 1 | 1 | 816.0889 | 35.6 | 0.00028 | VLFGEEEEQRQQEGVIVELSK |
| 3 | P25974 | 4218 | 50578 | 196 | 162 | 26 | 23 | 1 | 1 | 727.7091 | 61.18 | 7.60E-07 | IPAGTTYYLVNPHDHQNLK |
| 3 | P25974 | 512 | 50578 | 32 | 24 | 10 | 8 | 1 | 1 | 665.7886 | 58.05 | 1.60E-06 | EDENNPFYFR |
| 3 | P25974 | 2013 | 50578 | 125 | 90 | 23 | 20 | 1 | 1 | 618.3002 | 55.58 | 2.80E-06 | VLFGEEEEQR |
